# Activity of crizotinib over choroidal metastases in Non-Small-Cell Lung Cancer (NSCLC)-ALK rearranged: a case report

**DOI:** 10.1186/1756-0500-7-589

**Published:** 2014-09-02

**Authors:** Alessandra Bearz, Sandra Santarossa, Antonio Manfrè, Giorgio Beltrame, Martina Urbani, Ivana Sartor, Valentina Da Ros, Umberto Tirelli

**Affiliations:** Division of Medical Oncology A, Department of Medical Oncology, CRO Aviano, National Cancer Institute, Via Franco Gallini 2-33081, Aviano, PN Italy; Division of Ophthalmology, S. Maria degli Angeli Hospital, Pordenone, Italy; Division of Radiology, CRO Aviano, National Cancer Institute, Aviano, Italy; Clinical Trial Office, CRO Aviano, National Cancer Institute, Aviano, Italy

**Keywords:** NSCLC, Choroidal metastasis, ALK-translocation

## Abstract

**Background:**

Adenocarcinoma of the lung with EML4-ALK translocation is a rare subtype of Non Small-Cell Lung Cancer (NSCLC) that has recently shown to benefit from treatment with crizotinib. Despite the concerns about the efficacy of crizotinib over cerebral metastases, some reports have described its activity, although always after local treatment with radiotherapy. Recently it has been reported activity of crizotinib over choroidal metastases, again after radiotherapy.

**Case presentation:**

Herein we report a case of activity of crizotinib over choroidal metastases not previously treated with radiotherapy.

**Conclusion:**

We suggest crizotinib may be active over choroidal metastases in a patient harboring ALK translocation with no need of radiotherapy.

## Background

EML4-ALK-rearranged adenocarcinoma represents about 5-7% of Non Small Cell Lung Cancer (NSCLC). Treatment for patients affected by metastatic adenocarcinoma of the lung harbouring an EML4-ALK translocation is a novel oral compound anti MET known as crizotinib [1]. It is efficient on all sites of metastasis, except on cerebral secondary lesions: this is possibly due to a possible poor central nervous system (CNS) penetration.

Here we report a case of a patient with NSCLC adenocarcinoma, with ALK-rearrangement and bone, lymph nodal and choroidal metastases at diagnosis, who showed an impressive response to crizotinib in all sites.

## Case presentation

**Figure 1:**
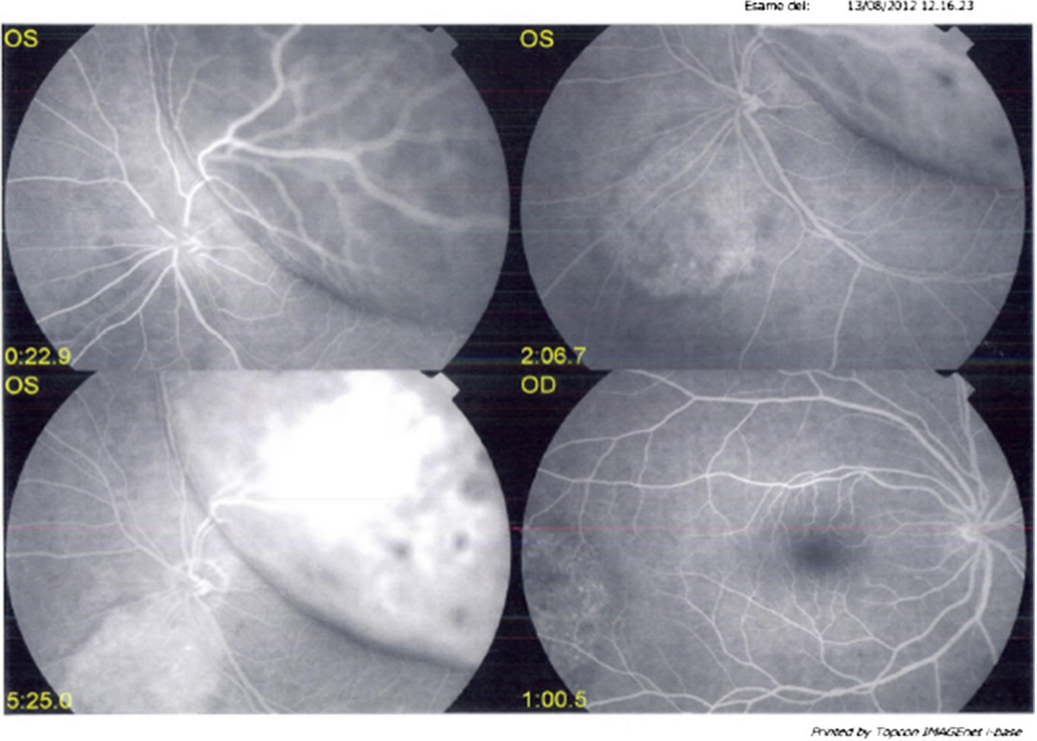
**Basal Fluorangiography, showing an extensive bullous essudative left retinal detachment.**

In August 2012, a 43-year-old man was hospitalized for the sudden onset of visual impairment (left eye). He reported not having had any traumas in the days before hospital admission, but referred having suffered for one month from headache, mostly located in the left eye area. He underwent a fluoroangiography, which showed an extensive bullous exudative left retinal detachment that involved the whole left retina, and was due to multiple, non-primitive, choroidal retinal neoplasias (Figure 1). Computed Tomography (CT)-scan and Magnetic Resonance Imaging of the brain showed the presence of abnormal tissue in the right retina and in most part of the left retina, where a clinically significant oedema was present.

Thorax CT-scan revealed a nodule in the right lung, in the median lobe, with the enlargement of right mediastinal lymph nodes. Bronchoscopy diagnosed an adenocarcinoma of the lung. Bone scan showed the involvement of several bones, cervical and dorsal vertebral bodies, and sternum. Biopsy of the right lung lesion was performed during bronchoscopy and an adenocarcinoma of the lung was diagnosed. Genetic characterization showed the presence of EGFR wild-type, KRAS wild-type, and EML4-ALK rearrangement which were diagnosed by FISH. Clinical TNM at the diagnosis was T2aN2M1, and distant localizations were in the bone and bilateral choroids mostly on the left eye.

**Figure 2:**
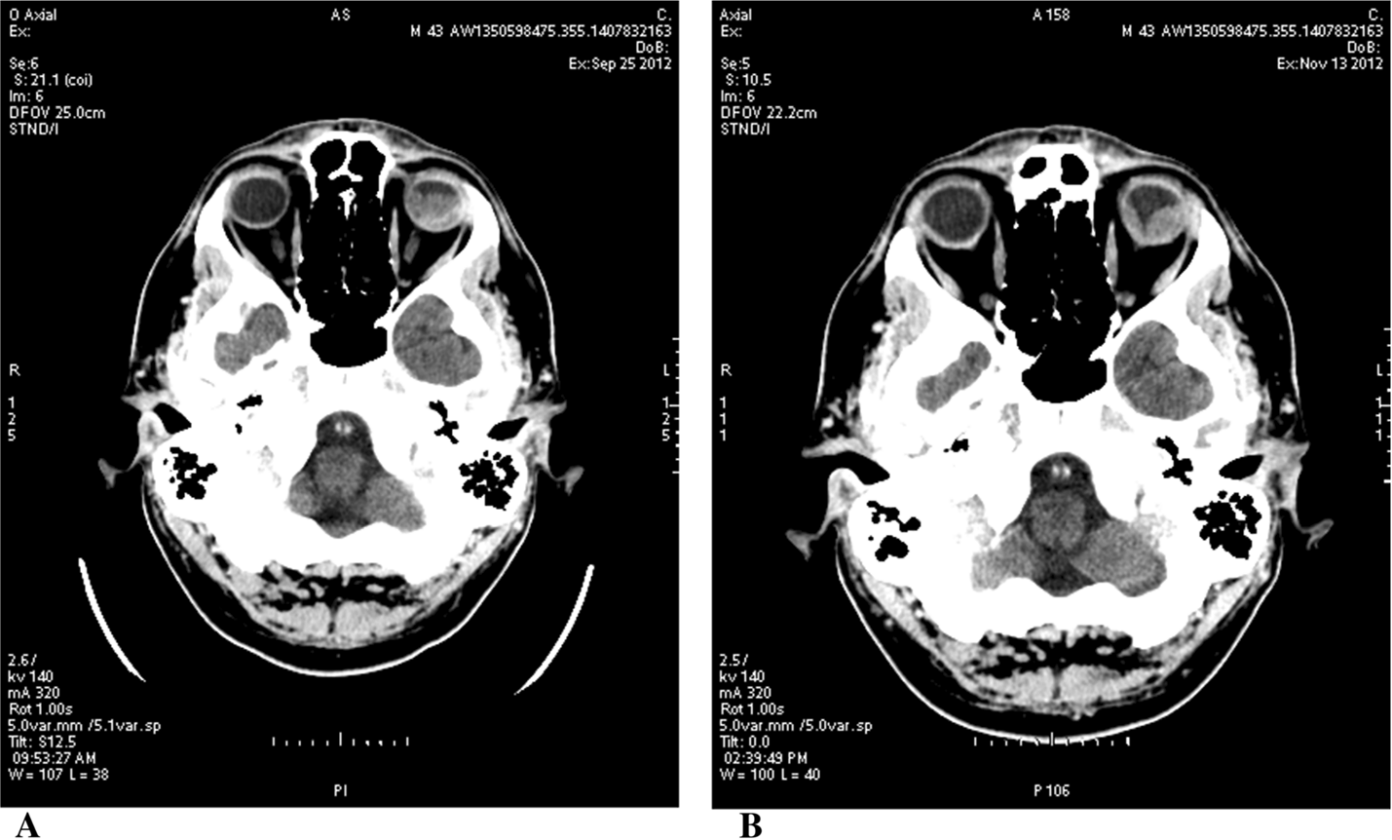
**Basal CT-scan of the eyes (A) and after 45 days on crizotinib (B), showing a reduction of the abnormal tissue in the left eye.**

**Figure 3:**
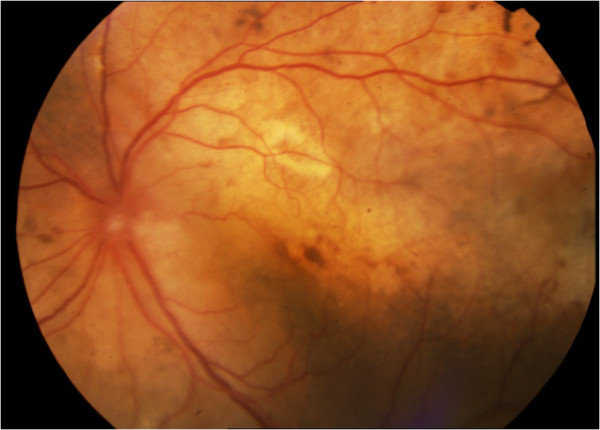
**Fluorangiography of the left eye, after 6 months in crizotinib and 2 months after phacoemulsification with lens implant.**

The patient reported pain in his head, neck, dorsal back region, and severe asthenia; he could only see a few lights in his lower left visual field. He had been staying in bed most of his daily time for one week. He started to receive crizotinib 250 mg BID orally at the beginning of October 2012. Treatment was well tolerated, with a few G1 episodes of diarrhoea.In a few weeks, symptoms improved with the resolution of pain and asthenia, and the patient could finally resume normal daily activities. Surprisingly, he reported an improvement in his left eye, as demonstrated by the comparison of basal CT-scan and CT-scan after 45 days of treatment (Figure 2), and at the end of January 2013 he was admitted to eye surgery. The patient underwent phacoemulsification with lens implant, 23 g vitrectomy and an infusion of perfluorocarbon liquid (PFCL) to smoothen the left retina. Endolaser was performed in the superior temporal retina area; finally, ocular tamponade with silicone oil 1000 cts was performed.In the following months, the retina remained adherent (Figure 3). However, the eyesight was limited to the lower sector of the visual field for the presence of exudative lower mass with no signs of expansion, as well as for the blurring caused by tamponade with silicone oil.

Nevertheless, patient started to drive again and began a new job, thus resuming his normal daily activities.

CT-scans showed partial regression of the right lung nodule, as well as of the mediastinal lymph nodes. Bone scan revealed reduction of the previously evidenced activity in the bone. After 15 months, CT-scans showed signs of progression: while choroidal metastases were stable, as well as bone metastasis, the nodule in the right lung and the mediastinal lymph nodes showed a progression of about 21% according to RECIST criteria. Patient stopped treatment with crizotinib and switched to a second-generation anti-ALK therapy (Alectinib, Roche), thus obtaining a partial response of the lung nodule and mediastinal lymph nodes two months later, still ongoing after 7 months, and stabilization of the choroidal metastases.

## Discussion and conclusions

Crizotinib is active against lung adenocarcinoma with EML4-ALK translocation. However, the median length of response duration is still unknown, as well as the efficacy of crizotinib in reaching central nervous tissue. Several reports showed the progression in central nervous tissue, claiming a possible inefficacy of crizotinib to overcome the central nervous barrier [1–3].

Nevertheless, at present there are only a few reports on the crizotinib response to CNS metastases in the literature [4, 5]. These cases had been pre-treated with radiotherapy: this could explain the increase in blood–brain barrier permeability, thus justifying the response, or it is likely there might be a different benefit of crizotinib in the CNS postiradiation as for other inhibitors [6]. There is one report about choroidal metastases responding to crizotinib, however, even in this case, both choroids had previously been treated with radiotherapy [7]. Our case describes for the first time a case of choroidal metastases responding to crizotinib without previous radiotherapy. Choroidal metastases were reduced with crizotinib and eyesight had improved. Our patient had never received any radiotherapy; at the moment of progression, the primary site tumor, e.g. the lung, had increased, while choroidal metastases were still stable.

The blood–retinal barrier is required to maintain the proper environment of neural retina controlling permeability through the choroid capillaries and working as a selective partition between the retina and the circulation [8]. Based on our experience, we suggest that crizotinib can reach choroidal metastases, possibly through the fenestrated endothelium of choroidal vessels.

## Consent

Written informed consent was obtained from the patient for publication of this Case report and any accompanying image. A copy of the written consent is available for review by the Editor of this journal.

## Abbreviations

NSCL: Non Small Cell Lung Cancer
CNS: Central nervous system
PFCL: Perfluorocarbon liquid
CT: Computed tomography
BRB: Blood–retinal barrier.
